# Task-Related Differences in End-Point Kinematics in School-Age Children with Typical Development

**DOI:** 10.3390/bs13070528

**Published:** 2023-06-23

**Authors:** Julia Mazzarella, Daniel Richie, Ajit M. W. Chaudhari, Eloisa Tudella, Colleen K. Spees, Jill C. Heathcock

**Affiliations:** 1 Division of Physical Therapy, School of Health and Rehabilitation Sciences, College of Medicine, The Ohio State University, Columbus, OH 43210, USAjill.heathcock@osumc.edu (J.C.H.); 2 Department of Biomedical Engineering, College of Engineering, The Ohio State University, Columbus, OH 43210, USA; 3 Department of Physical Therapy, Federal University of São Carlos, São Carlos 13565-905, SP, Brazil; 4 Division of Medical Dietetics, School of Health and Rehabilitation Sciences, College of Medicine, The Ohio State University, Columbus, OH 43210, USA

**Keywords:** upper extremity, technology, pediatric, functional reach, assessment

## Abstract

Understanding whether and how children with typical development adapt their reaches for different functional tasks could inform a more targeted design of rehabilitation interventions to improve upper extremity function in children with motor disabilities. This prospective study compares timing and coordination of a reach-to-drink, reach-to-eat, and a bilateral reaching task in typically developing school-aged children. Average speed, straightness, and smoothness of hand movements were measured in a convenience sample of 71 children, mean age 8.77 ± 0.48 years. Linear mixed models for repeated measures compared the variables by task, phases of the reach, task x phase interactions, and dominant versus non-dominant hands. There were significant main effects for task and phase, significant task x phase interactions (*p* < 0.05), and a significant difference between the dominant and non-dominant hand for straightness. Hand movements were fastest and smoothest for the reach-to-eat task, and least straight for the bilateral reaching task. Hand movements were also straighter in the object transport phases than the prehension and withdrawal phases. These results indicate that children with typical development change their timing and coordination of reach based on the task they are performing. These results can inform the design of rehabilitation interventions targeting arm and hand function.

## 1. Introduction

Functional reach is essential for children to complete tasks like feeding, self-care, school participation, and leisure activities. Intentional reach is essential for driving other areas of development, such as cognitive and fine motor, in young children. Intentional reach-to-grasp skills emerge between 4–6 months of age in typical development as infants begin reaching for toys [1,2] and reach adult-like performance by 8–11 years [3,4,5]. Reaching and grasping are challenging skills for many children with motor disabilities, such as cerebral palsy (CP), and often a major focus of rehabilitation. The most common measures of functional upper extremity use in this population apply clinical observation and subjective scoring of performance, which have limitations in precision and rater bias [6,7]. Kinematic variables collected using three-dimensional (3D) motion capture offer an objective and quantitative measurement of upper extremity movements in early child development [3,8,9,10,11,12,13,14,15,16] such as reach and grasp. Timing and coordination of reach and grasp in infants and children are well-documented in the literature [3,5,9,14,16,17,18,19,20].

When quantifying upper extremity movement, a reach is typically defined as a movement towards an object, ending when the hand contacts the object. Straightness and smoothness are variables that quantify the coordination of a reach [17]. Straightness describes the trajectory of the hand, with a straighter movement following a shorter trajectory from the start- to end-point of the movement. Smoothness describes the shape of the velocity profile of the hand, with fewer peaks in the velocity profile indicating a smoother movement. A volitional reach in a healthy adult typically presents with a single velocity peak and a near-straight trajectory with a single, shallow curve [18]. Speed of the reach is calculated to measure timing of the reach movement and both average velocity and peak velocity are frequently used. Speed of a reach increases with maturity [3].

In the past decade, a few studies have applied 3D kinematics to objectively measure reach in a functional task, such as reaching for food to eat or a cup to drink [17,19,20,21]. Butler et al. [17,20] published the Reach and Grasp Cycle, a protocol developed to objectively measure upper extremity movement during a functional reach-to-drink task. Butler et al. [17,20] and Machado et al. [19] have applied this protocol in samples of children with typical development and children with cerebral palsy with upper extremity motor impairments. Hung et al. [21] used a similar reach-grasp-eat task, which involved reaching to eat a cracker, to evaluate functional upper extremity movement in children with CP. These studies provide detailed information about reach and grasp during discrete functional tasks in populations of children with typical development and CP. They did not compare differences in reach kinematics with the type of task and use of the dominant versus non-dominant upper extremity. Previous research in adults has investigated differences in kinematics when reaching for different objects; however, these did not include functional, multi-phase reaching tasks [22,23,24]. This study aims to fill the gap in understanding about how children with typical development change their reaches for different functional tasks. This will inform more precise treatment planning for the improvement of functional upper extremity use and coordination in children with motor impairments.

We adapted the set-up, procedure, and movement sequence from the Reach and Grasp Cycle [20] to three different tasks: a reach-to-drink, reach-to-eat, and a bilateral reach. Our purpose was to measure kinematics of functional reach in typically developing school-aged children when performing various everyday tasks to identify differences in spatiotemporal characteristics of reach. We measured straightness, smoothness, and average speed of hand movement for each phase of the Reach and Grasp Cycle. We hypothesized that there would be significant differences in all three kinematic variables by type of task, phases of the Reach and Grasp Cycle, and between the dominant and non-dominant hands for unilateral tasks. Specifically, we expected the initial reach (prehension) to be less straight, less smooth, and slower than the other 3 phases. We expected movements in the first 3 phases to be straighter, smoother, and slower in the reach-to-drink task compared to the other two tasks. We also expected movements to be less straight, less smooth, and slower in the bilateral reach, due to the bilateral coordination aspect. Last, we expected movements with the dominant hand to be straighter, smoother, and faster than with the non-dominant hand.

## 2. Materials and Methods

### 2.1. Participants

Participants were 71 typically developing children (38 male, 33 female), 7–10 years old (mean 8.77 ± 0.48), with 10 left-hand dominant and 61 right-hand dominant, as determined by the child’s preferred writing hand. Participants were a convenience sample of children recruited from low-resource neighborhoods in Central Ohio, USA. Children were excluded if they were unable to follow instructions given in English, or if they had a motor impairment that prevented them from being able to perform the reach and grasp tasks. Given the exploratory nature of this investigation, the sample size was determined based on recommendations for normative datasets in pediatric populations [25].

This study was approved by the Institutional Review Board at The Ohio State University (2017B0110). Parents or legal guardians provided informed consent, and all child participants provided verbal assent.

### 2.2. Procedure

**Figure 1:**
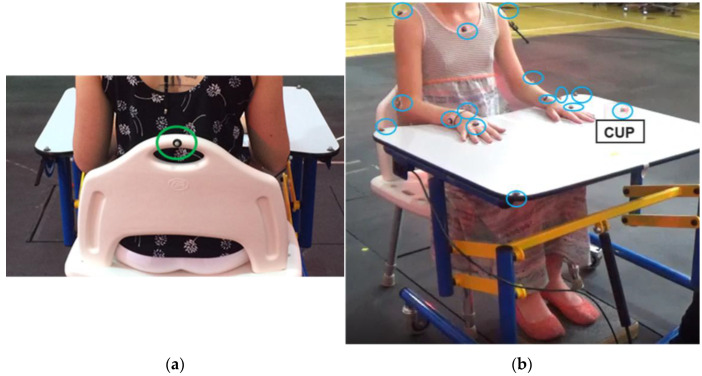
Marker placement and child positioning; (**a**) view from behind; (**b**) view from front.

Participants were assessed at the Pediatric and Rehabilitation (PEARL) Laboratory at The Ohio State University. Retroreflective markers (8 mm) were placed on the children on their sternum, bilateral acromia, lateral epicondyles, radial and ulnar styloid processes, and heads of the third metacarpals. Markers were also placed on the 4 corners of the table, the back of the chair, and the objects that the children were reaching for (Figure 1). Children were seated at the table with the chair positioned so that their hips, knees, and ankles were flexed to 90°. The objects for which children were reaching (a 5.6 cm diameter cup of water, a 4.6 cm diameter Ritz cracker, or a 21.6 cm diameter ball) were positioned in front of the child at 75% of their arm’s length away, consistent with the set-up from Butler et al. and Machado et al. [19,20]. This position was marked with tape on the table for each participant. The participants began with their hands resting on the table, shoulders neutral, elbows flexed to 90°, and wrists neutral. The resting hand placement was marked on the table with hand-shaped outlines, within which the children placed their hands. For the reach-to-drink and reach-to-eat tasks, children were instructed: “With your [left/right] hand, reach for the [object], pick up the [object], take a [drink/bite], return the [object] to the marked position on the table, and return your hand to the start position. Do this twice.” For the bilateral task, the children were instructed: “Reach for the ball with both hands, pick it up, touch it to your chin, return the ball to the marked position, and return your hands to the start position. Do this twice.” Participants were allowed one practice trial per hand with each object. Two repetitions were recorded for each condition with a 10-camera VICON Motion Capture system at 120 Hz and filtered with a low-pass Butterworth filter at 4 Hz.

### 2.3. Data Analysis

**Figure 2:**
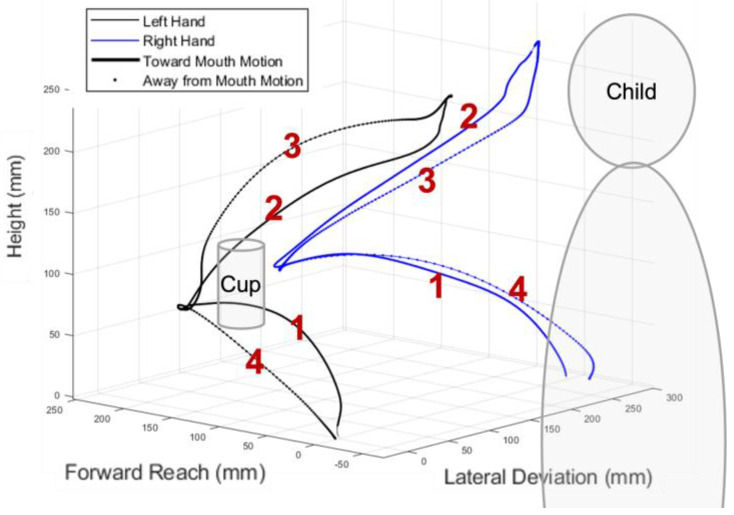
Four phases of the reaching task: (1) Prehension; (2) Transport 1; (3) Transport 2; (4) Withdrawal.

Phases of the movement (Figure 2) were visually identified and marked as events in the Vicon recordings. The task was divided into the following 4 phases of movement, based on the Reach and Grasp Cycle created by Butler et al. [20]:

1. Prehension: The movement begins with the hand(s) in the marked start position and ends when the hand(s) contact the object.
2. Transport 1: The movement begins with the hand(s) lifting the object from the table and ends with the object contacting the mouth or chin.
3. Transport 2: The movement begins with the object leaving the mouth or chin and ends with the object contacting the table at its marked position.
4. Withdrawal: The movement begins with the hand(s) releasing the object and ends with the hands contacting the marked start position on the table.

A custom MATLAB script was used to calculate spatial and temporal variables of reach for each phase of the Reach and Grasp Cycle. The variables were calculated based on the hand position, which was defined as the center point between the markers on the 3rd metacarpal, ulnar styloid process, and radial styloid process. The spatial and temporal variables were defined as:

- Straightness (ratio): ratio of the hand path length (total path the hand travels) to the movement length (difference between start- and end-points of the movement), with a value closer to one indicating a straighter movement.
- Smoothness (count): measured as the number of velocity peaks in a movement, with fewer velocity peaks indicating a smoother movement.
- Average speed (mm/s): calculated at each position of the marker using a 4-point central difference numerical differentiation.

A generalized linear mixed model for repeated measures for Gaussian distributions was applied using the SAS GLIMMIX procedure to compare each kinematic variable by task, phase, task x phase interactions, and dominant versus non-dominant hand for unilateral tasks (reach-to-drink and reach-to-eat). The model was created using a restricted maximum likelihood estimation technique, and the Kenward–Roger degrees of freedom method and fixed effects standard error adjustment. The alpha level was set to α = 0.05. This model was chosen because it accounts for correlations between dependent variables, as well as a response that is not normally distributed. This is the case for straightness ratio and smoothness, for which values can only be >1. Least squared means were calculated for post hoc testing of task x phase interactions. The Bonferroni correction was applied for post hoc comparisons, setting α = 0.0005.

## 3. Results

Means and standard deviation for kinematic variables of interest by task, type, and phase were calculated (Table 1).

### 3.1. Straightness Ratio

**Figure 3:**
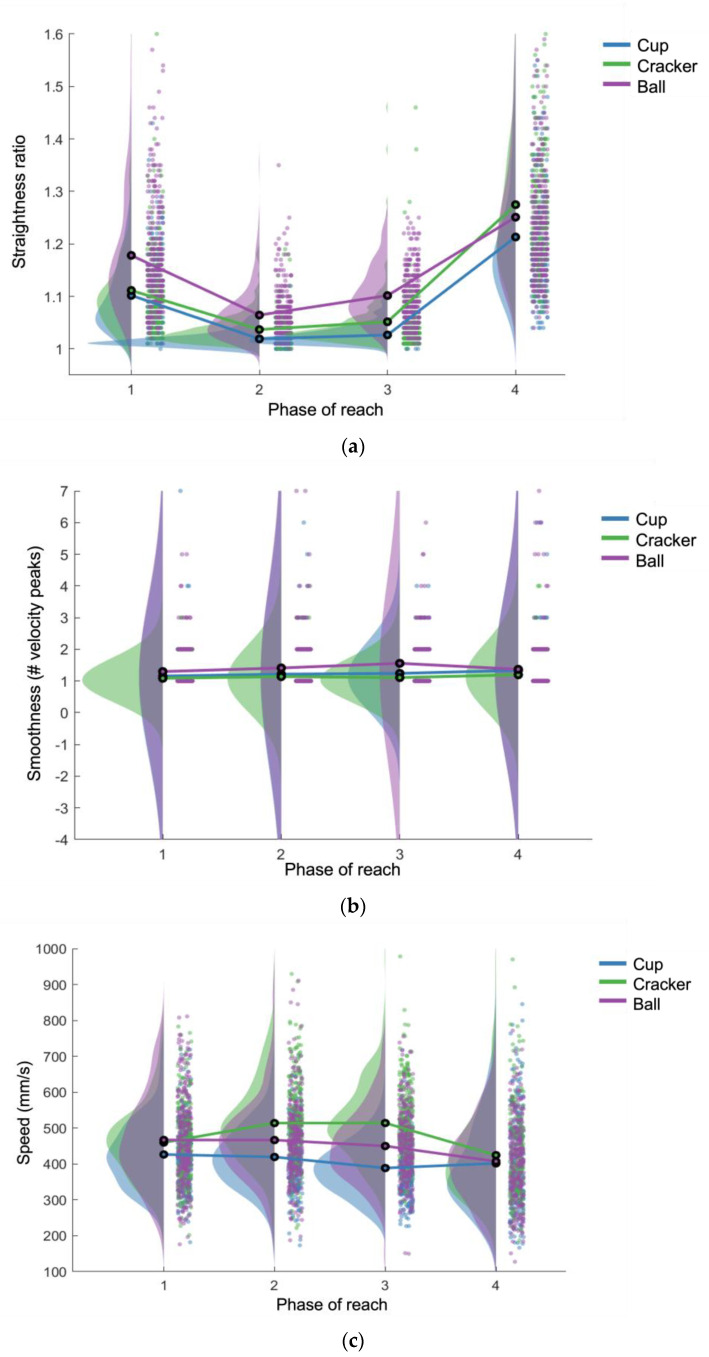
Raincloud plot by task and phase of straightness ratio (**a**), smoothness (**b**), and speed (**c**) [26].

There were significant main effects of straightness ratio for type of task (F(2) = 107.26, *p* < 0.0001), phase of task (F(3) = 801.98, *p* < 0.0001), type x phase interaction (F(6) = 12.59, *p* < 0.0001), and hand dominance (F(1) = 13.05, *p* = 0.0003), with straighter movement on the dominant side (Table 2; Figure 3a). Post hoc analysis revealed significant differences (*p* < 0.0005) for all comparisons, except: the prehension, transport 1, and transport 2 phases between the reach-to-drink versus the reach-to-eat tasks; the transport 1 and withdrawal phases between the bilateral versus reach-to-eat tasks; and within the reach-to-drink and reach-to-eat tasks, the transport 1 versus transport 2 phases; these comparisons showed no significant differences (Table 3).

### 3.2. Smoothness

There was a significant main effect of smoothness for type of task (F(2) = 21.06, *p* < 0.0001). Main effects for phase, type x phase interaction, and dominant versus non-dominant hand were not significant (Table 2; Figure 3b). Post hoc analysis revealed that the reaching movements for the bilateral task were significantly less smooth than in the reach-to-eat task for transport 2 (*p* < 0.0005). The reaching movement for transport 2 was also significantly less smooth than withdrawal for the bilateral task (*p* = 0.0005). All other comparisons were not significantly different (Table 3).

### 3.3. Speed

There were significant main effects of speed for type of task (F(2) = 176.31, *p* < 0.0001), phase of task (F(3) = 64.87, *p* < 0.0001), and type x phase interaction (F(6) = 25.38, *p* < 0.0001), but not for dominant versus non-dominant hand (Table 2; Figure 3c). Post hoc testing revealed the following phase differences within each task: reaching movements in the prehension phase were faster than transport 2 and withdrawal, and reaching movements in the transport 1 phase were faster than transport 2; for the reach-to-eat task, reaching movements were faster in the prehension phase than the three other phases, and withdrawal was slower than all three other phases; reaching movements in the bilateral reach were significantly slower in the withdrawal phase than all other phases (Table 3). The following differences between tasks by phase were also found: for prehension and transport 1, reach movement was faster in both the bilateral and reach-to-eat tasks versus the reach-to-drink task; reaching movement in the reach-to-eat task was also significantly faster than the bilateral task for both transport phases.

## 4. Discussion

The objective of this study was to evaluate the coordination and timing of reaching movements in typically developing school-aged children when performing functional reaching tasks, applying the Reach and Grasp Cycle [20]. We were able to measure the straightness, smoothness, and speed of reaching movements of 71 children performing three different tasks: reach-to-drink, reach-to-eat, and a bilateral reach. The results from this study support our main hypothesis, indicating that reaching movement varies significantly based on the task being performed, even when the individual is following the same reaching pattern of the Reach and Grasp Cycle [20]. Support for our specific hypotheses was mixed. The prehension movement was slower and straighter than the two transport phases, as predicted, but not the withdrawal phase. Additionally, there was no significant difference in smoothness between the phases. This might have been due to a ceiling effect, as the mean was close to 1 for all phases. As we expected, bilateral movements were less straight than the other two tasks. The bilateral movements were also slower than the reach-to-eat movements, but not reach-to-drink, providing partial support for that hypothesis. Last, the dominant hand did move significantly straighter than the non-dominant hand, but not smoother nor faster. These results highlight the complex interplay of task and object characteristics that impact the timing and coordination of reaching behaviors in children. Since most of the research on the impacts of varying task and object constraints on reach kinematics has focused on an adult population, we will compare and contrast our results with those.

Research in adults has demonstrated changes in reaching behaviors with different task constraints. For example, when adults reach for an empty cup of water, their movements are significantly faster than reaches toward a full cup of water [24,27]. It is not yet known if these effects are the same in a pediatric population. In support of our hypotheses, some of the results in this study were consistent with these findings, such as: reaches were faster in the prehension and transport 1 phases for the reach-to-eat and bilateral tasks than for the reach-to-drink task. This is likely due to greater constraints on the reach-to-drink task, with the need for precision to keep the water in the cup. Consistent with this theory, movements in the two transport phases were straightest for the reach-to-drink task, although only significantly different from the bilateral task. This type of task requires feedback motor control to maintain a straight movement to keep the water from spilling out of the cup [23], meaning that the sensory systems are providing real-time feedback on the position of the cup, water, hand, etc., which the motor system then uses to guide the movement of the upper extremity to complete the task successfully. The children likely slowed their speed in the reach-to-drink task to allow time for this sensory processing to occur.

Changes in object size also affects reach speed in adults performing a reach and grasp task, with slower reaches toward smaller objects [23,28]. In contrast, children in this study had the fastest reaching speeds in the reach-to-eat task, even though the cracker was the smallest of the three objects. In this instance, there are likely other factors that had greater influence on reach behavior than object size. One factor might be that reaching for a cup full of water requires more precision and visuomotor feedback than reaching for a small object, as described previously. An additional factor could be motivation to complete the task. Motivation and reward can impact movement speed, amplitude, and variability in multiple types of movement tasks in adults [22,29,30,31]. In the present study, it is possible that the children found the reward of eating a cracker to be more motivating than drinking water or touching a ball to their chin. This would be consistent with previous research, which indicated that reaching movements were faster and less variable for tasks that offered a reward, compared to tasks with no reward [22,29].

The differences between phases of the Reach and Grasp Cycle in this study were partially consistent with our hypotheses and might indicate that familiarity with specific movements has a strong influence on timing and coordination of the reach. For example, reaches were fastest and straightest in the object transport phases, which is consistent with previous research and our predictions [17,32]. It is possible that these movements, or at least transport 1, can be completed using primarily feedforward control, which is much faster than feedback control [33]. Feedforward motor control relies on stored motor representations, which are created over many repetitions of a motor task, rather than from processing of sensory information in real time. Children bring objects to and from their mouth many times a day; however, the position of the object they are reaching for is variable, and thus the prehension phase requires more feedback control to accomplish. We had also hypothesized that movements would be faster and straighter in the withdrawal phase compared to the prehension phase, which was not supported by our results. It is possible that in the context of this study procedure, participants needed to use feedback control for the withdrawal phase as well, because there was a marked position on which they needed to place their hands. Overall, it is clear that there is a complex interplay of task and object characteristics that impact timing and coordination of reaching behaviors in children via motor control mechanisms.

Last, we found that the dominant hand movements were significantly straighter than the non-dominant hand movements, as we expected. Contrary to our hypothesis, and contrary to studies in adults, we found no differences in speed or smoothness of movement between the dominant and non-dominant hands [34,35]. Given the age and typical development of the participants (7–12 years old), we would expect them to demonstrate a strong hand preference, and our result partially supported this [36]. It is possible that this procedure is not sensitive enough to detect the difference in speed and smoothness between the dominant and non-dominant hands in typical development; however, Butler et al. have shown that it can detect differences between typical and motor-impaired movement [17]. Future investigations should determine the magnitude of difference required to detect a meaningful change for each of these kinematic variables in the Reach and Grasp Cycle. This would help determine whether measuring kinematics of functional reaching tasks could be used to detect clinically important improvements with rehabilitation.

## 5. Conclusions

Kinematic measurement of coordination and timing of reaching movement in functional reaching tasks is a promising objective, quantitative measure of upper extremity function in a pediatric population, as it can provide detailed information about movement timing and coordination. Results from this study provide insight into how task familiarity, constraints, and motivation might impact the coordination and timing of reach in typically developing children, and the role that motor control systems might play in those differences. This information could be used to further refine clinical rehabilitation approaches for pediatric populations with upper extremity motor impairment. Application to clinical practice could include: (1) routinely using kinematics as an outcome measure to track progress; (2) using information about timing and coordination and task constraints to target improvements in skills that are inherent to participation in daily life activities. Some examples of precise treatment planning based on the results of this study would be to choose larger target objects or more motivating tasks to train increased reach speed, and to use tasks that require feedback control, such as transporting a cup of water, to train straightness and smoothness. Future research could further investigate the impact of specific task characteristics on kinematics in a reach and grasp task in children. An important next step would be to determine a minimal clinically important difference for average speed, smoothness, and straightness of hand movement in the Reach and Grasp Cycle. In the future, data could be used as a normative comparison for age-matched children with motor disability to measure improvements in everyday reaching function with targeted upper extremity therapy interventions.

## Figures and Tables

**Table 1:** Mean and standard deviation of straightness ratio, smoothness, and average speed of hand movement by task type and phase.

Task Type	Phase	Variable	Mean	Std. Deviation
Bilateral	Prehension	Straightness Ratio	1.18	0.109
		Smoothness	1.70	3.59
		Speed (mm/s)	465	125
	Transport 1	Straightness Ratio	1.06	0.049
		Smoothness	1.75	2.68
		Speed (mm/s)	467	113
	Transport 2	Straightness Ratio	1.10	0.0528
		Smoothness	1.97	3.44
		Speed (mm/s)	450	99.9
	Withdrawal	Straightness Ratio	1.25	0.150
		Smoothness	1.43	1.31
		Speed (mm/s)	407	114
Reach-to-eat	Prehension	Straightness Ratio	1.11	0.0792
		Smoothness	1.10	0.357
		Speed (mm/s)	460	104
	Transport 1	Straightness Ratio	1.04	0.0294
		Smoothness	1.14	0.415
		Speed (mm/s)	514	116
	Transport 2	Straightness Ratio	1.05	0.0799
		Smoothness	1.11	0.345
		Speed (mm/s)	514	105
	Withdrawal	Straightness Ratio	1.28	0.171
		Smoothness	1.18	0.435
		Speed (mm/s)	423	121
Reach-to-drink	Prehension	Straightness Ratio	1.10	0.0692
		Smoothness	1.19	0.852
		Speed (mm/s)	429	93.6
	Transport 1	Straightness Ratio	1.02	0.0174
		Smoothness	1.34	1.70
		Speed (mm/s)	420	87.4
	Transport 2	Straightness Ratio	1.03	0.0185
		Smoothness	1.48	2.07
		Speed (mm/s)	389	76.3
	Withdrawal	Straightness Ratio	1.21	0.175
		Smoothness	1.33	0.807
		Speed (mm/s)	399	128

**Table 2:** Main effects for least squares means.

		Straightness Ratio			Smoothness			Speed		
Effect	Num DF	Den DF	F	*p*-Value	Den DF	F	*p*-Value	Den DF	F	*p*-Value
Task	2	3124	107.26	<0.0001 *	3122	21.06	<0.0001 *	3112	176.31	<0.0001 *
Phase	3	3113	801.98	<0.0001 *	3114	2.26	0.0798	3109	64.87	<0.0001 *
Task x Phase	6	3111	12.59	<0.0001 *	3113	1.68	0.1230	3109	25.38	<0.0001 *
Hand	1	3124	13.05	0.0003	3122	1.03	0.3107	3112	0.18	0.6674

* Significant effect.

**Table 3:** Differences of task x phase least squares means.

				Straightness Ratio		Smoothness		Speed	
Task	Phase	Task	Phase	*t* Value	*p*-Value	*t* Value	*p*-Value	*t* Value	*p*-Value
Ball	1	Ball	2	14.88	<0.0001 *	−0.31	0.7596	−0.33	0.7386
Ball	1	Ball	3	10.06	<0.0001 *	−2.02	0.0438	2.11	0.0352
Ball	1	Ball	4	−9.02	<0.0001 *	1.55	0.1208	8.13	<0.0001 *
Ball	2	Ball	3	−4.75	<0.0001 *	−1.69	0.0902	2.42	0.0158
Ball	2	Ball	4	−23.44	<0.0001 *	1.84	0.0664	8.39	<0.0001 *
Ball	3	Ball	4	−18.73	<0.0001 *	3.50	0.0005 *	6.00	<0.0001 *
Cracker	1	Cracker	2	9.71	<0.0001 *	−0.25	0.8026	−7.60	<0.0001 *
Cracker	1	Cracker	3	7.84	<0.0001 *	−0.04	0.9664	−7.63	<0.0001 *
Cracker	1	Cracker	4	−19.94	<0.0001 *	−0.76	0.4476	4.82	<0.0001 *
Cracker	2	Cracker	3	−1.86	0.0626	0.21	0.8365	−0.03	0.9773
Cracker	2	Cracker	4	−29.31	<0.0001 *	−0.51	0.6102	12.22	<0.0001 *
Cracker	3	Cracker	4	−27.47	<0.0001 *	−0.71	0.4757	12.25	<0.0001 *
Cup	1	Cup	2	10.72	<0.0001 *	−1.04	0.2992	1.17	0.2426
Cup	1	Cup	3	9.78	<0.0001 *	−1.96	0.0499	5.52	<0.0001 *
Cup	1	Cup	4	−13.65	<0.0001 *	−0.92	0.3589	3.74	0.0002 *
Cup	2	Cup	3	−0.93	0.3546	−0.92	0.3585	4.33	<0.0001 *
Cup	2	Cup	4	−24.13	<0.0001 *	0.11	0.9129	2.57	0.0102
Cup	3	Cup	4	−23.21	<0.0001 *	1.02	0.3085	−1.71	0.0868
Ball	1	Cracker	1	8.41	<0.0001 *	3.40	0.0007	0.68	0.4961
Ball	1	Cup	1	9.75	<0.0001 *	2.78	0.0054	5.06	<0.0001 *
Cracker	1	Cup	1	1.29	0.1987	−0.62	0.5331	4.33	<0.0001 *
Ball	2	Cracker	2	3.41	0.0007	3.39	0.0007	−6.60	<0.0001 *
Ball	2	Cup	2	5.62	<0.0001 *	2.00	0.0452	6.47	<0.0001 *
Cracker	2	Cup	2	2.17	0.0301	−1.39	0.1641	13.00	<0.0001 *
Ball	3	Cracker	3	6.22	<0.0001 *	5.26	<0.0001 *	−9.01	<0.0001 *
Ball	3	Cup	3	9.40	<0.0001 *	2.77	0.0057	8.39	<0.0001 *
Cracker	3	Cup	3	3.12	0.0018	−2.51	0.0121	17.33	<0.0001 *
Ball	4	Cracker	4	−2.91	0.0037	0.99	0.3226	−2.46	0.0139
Ball	4	Cup	4	4.76	<0.0001 *	0.25	0.8019	0.58	0.5595
Cracker	4	Cup	4	7.65	<0.0001 *	−0.74	0.4569	3.05	0.0023

* Significant effect.

## Data Availability

The data presented in this study are available on request from the corresponding author.
